# Relationship between endothelin and nitric oxide pathways in the onset and maintenance of hypertension in children and adolescents

**DOI:** 10.1007/s00467-021-05144-2

**Published:** 2021-06-03

**Authors:** Simonetta Genovesi, Marco Giussani, Antonina Orlando, Giulia Lieti, Francesca Viazzi, Gianfranco Parati

**Affiliations:** 1 School of Medicine and Surgery, University Milano - Bicocca, Milan, Italy; 2 Department of Cardiovascular, Neural, and Metabolic Sciences, S Luca Hospital, IRCCS, Istituto Auxologico Italiano, Milan, Italy; 3 Department of Internal Medicine, University of Study and IRCCS Ospedale Policlinico San Martino, Genoa, Italy

**Keywords:** Adolescents, Children, Endothelin-1, Hypertension, Nitric oxide, Obesity

## Abstract

The mechanisms that regulate blood pressure are numerous and complex; one mechanism that plays an important role in this scenario is represented by the balance between the vasoconstrictor effect of endothelin-1 and the vasodilator effect of nitric oxide. While there is agreement on the fact that increased endothelin-1 activity and decreased nitric oxide bioavailability are present in hypertensive adults, the situation is less clear in children and adolescents. Not all studies agree on the finding of an increase in plasma endothelin-1 levels in hypertensive children and adolescents; in addition, the picture is often confused by the concomitant presence of obesity, a condition that stimulates the production of endothelin-1. Furthermore, there is recent evidence that, in younger obese and hypertensive subjects, there is an overproduction of nitric oxide, rather than a reduction. This condition may change over time, causing endothelial dysfunction due to a reduced availability of nitric oxide in hypertensive adolescents. The purpose of this review is to address the main biochemical and pathophysiological aspects of endothelin and nitric oxide involvement in hypertension and to summarize the available scientific evidence on their role in the onset and maintenance of high blood pressure in children and adolescents.

## Introduction

It is widely known that blood pressure (BP) levels ​are the result of complex interactions between various organs and systems: the central and peripheral nervous system, the kidney, and the endocrine system. Recently, evidence has been provided that even at the level of the endothelium and vascular smooth muscle cells, autocrine or paracrine control mechanisms are actively involved in regulating vascular tone through a balance between substances either with vasoconstrictor or vasodilator action. Proper endothelial function is of fundamental importance in maintaining adequate BP levels. Although the biochemical factors involved in these regulatory mechanisms are numerous, endothelin (ET)-1, a substance with a prevalent vasoconstrictive effect, and nitric oxide (NO), which has an important vasodilating effect, are major players in this scenario. In addition to the action of these two substances, the role of endothelium-derived hyperpolarizing factors and of prostaglandins should also be considered. One of the possible causes contributing to the onset and maintenance of arterial hypertension, as early as in childhood, may be the imbalance between the activity of these substances. On the other hand, the presence of arterial hypertension may in turn have a harmful effect on endothelial function.

The prevalence of high BP in children and adolescents is not a negligible phenomenon [1], and it has become more clear in recent years that it is mainly due to essential (primary) hypertension cases [2]. As a high percentage of children and adolescents with arterial hypertension are overweight or obese [3], it can be assumed that excess weight is one of the main risk factors for the development of arterial hypertension in this age group.

The purpose of this educational review is to address the role of the most important substances involved in the regulation of endothelial function from a biochemical and pathophysiological point of view. Subsequently, the main clinical studies suggesting a relationship between endothelial dysfunction and arterial hypertension in children are presented.

## Endothelin

**Fig. 1:**
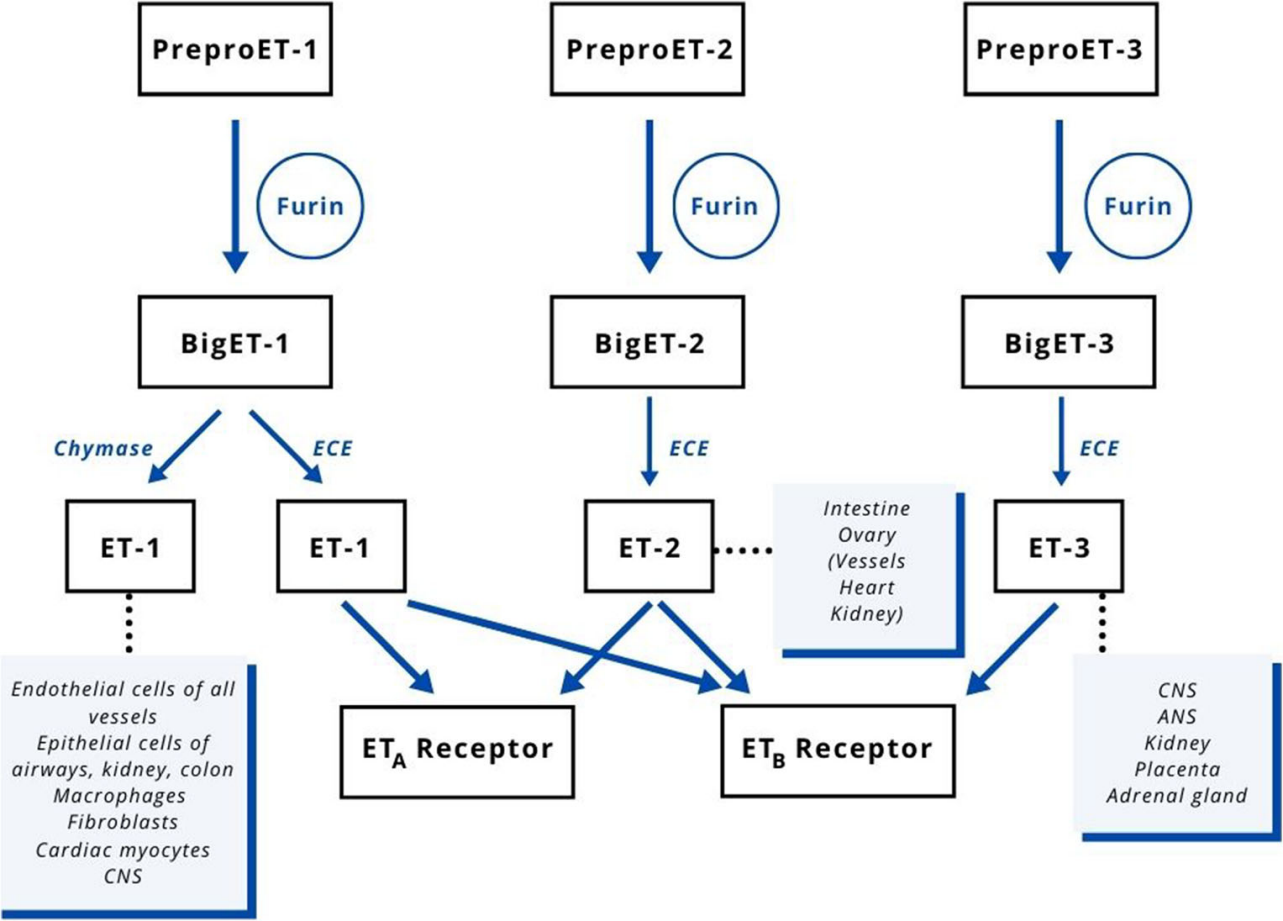
Activation of endothelin and interaction with receptors. ANS, autonomic nervous system; CNS, central nervous system; ECE, endothelin-converting enzyme; ET, endothelin

Endothelin (ET) is a family of multifunctional peptides, which play an important role in the physiology and pathology of both humans and other mammals. The name ET was first used in 1985 when a substance with an important vasoconstrictive activity was isolated from porcine endothelium cells for the first time [4–6]. Later, it was found that many other types of cells possess the ability to produce endothelin [7]. Endothelins consist of 21 amino acids with a hydrophobic C-terminal part, folded in the N-terminal part due to the presence of two disulfide bridges between two cysteine ​molecules. The three known isoforms of endothelin differ from each other only by the replacement of some amino acids [8]. Endothelins have a structure that is very similar to that of saraphotoxins, a family of poisons of some snakes. It is interesting to note how, in the course of evolution, probably starting from a single peptide common to both species, an exocrine poison has developed in reptiles, while substances with an autocrine or paracrine regulatory role have developed in mammals [9]. The currently known ETs are three, called ET-1, ET-2, and ET-3 [8, 10]. These three ETs are encoded by three different genes that give rise to three different precursor polypeptides called pre-proendothelin, which are first cleaved by a non-specific protease (furin) to form big-ET and then, by specific ET-converting enzymes (ECEs), undergo further hydrolysis to give rise to the active forms [11]. In humans, there are four isoforms of ECEs, which differ only in the N-terminal part and which derive from the same gene with different splicing in the transcription of the mRNA: ECE-1a, ECE-1b, ECE-1c, ECE-1d. Three of these isoforms are expressed on the cell surface, while ECE-1b is localized inside the cells near the Golgi apparatus [12]. Besides being produced through the action of ECEs, ET-1 can also be generated by the enzyme chymase [4]. At present, two ET receptors are known, ET_A_ and ET_B_. Endothelin ET-1 and ET-2 are most probably active on both receptors, while ET-3 exerts its effect only on the ET_B_ receptor [13, 14] (Fig. 1). Based on these findings, it has been possible to produce agonists and antagonists (with different levels of selectivity) of the two receptors, as well as ECE blockers. The experimental use of these substances has provided an important contribution to understanding the complex, and still not fully investigated, activity of the endothelin system. In general, the activation of the ET_A_ receptors promotes vasoconstriction, inflammation, and cell proliferation, while the ET_B_ receptors could be considered antagonists of all these actions. However, it is not certain that all observations made in the laboratory setting reflect exactly what actually happens in vivo [4]. In addition to the actions of interest for this review, it has been suggested that ET may also have an important role in the growth and development of organogenesis and that the activities of the three different ETs affect numerous organs and systems [15, 16], sometimes with synergistic and sometimes with antagonistic actions. Even though these peptides have been known for several years, the related research field is still largely to be explored.

## Endothelin ET-1

Endothelin-1 was the first ET to be discovered and is the most common endothelin in the human body. Endothelin-1 is produced in many tissues: in endothelial cells of both arteries and veins and in epithelial cells of the airways, kidney and colon. It is also present in macrophages, fibroblasts, cardiac myocytes, mesangial cells, enteric cells of the glia, podocytes, and neurons of the central nervous system [7]. Its main function is to favor the perfusion of organs by contributing to the maintenance of vascular tone through its vasoconstrictor activity. It is thought that the release of ET-1 from endothelial cells occurs in two ways: by continuous secretion of the substance in small vesicles, which ensures basic muscle tone, and by phasic secretion in larger vesicles occurring in response to different stimuli, both physical and chemical [17]. The main physical stimulus that leads to the release of ET-1 is a moderate level of vascular stress (shear stress). However, if the shear stress increases in an excessive manner, the production of ET-1 stops. Hypoxia is one of the most important chemical stimuli of ET-1 release. An increase in ET-1 synthesis is also observed, however, in response to growth factors and cytokines, such as thrombin, tumor necrosis factor-α, interleukin-1, and insulin, but also to vasoactive substances such as norepinephrine, angiotensin II, vasopressin, and bradykinin. In pediatric idiopathic pulmonary arterial hypertension, plasma ET-1 levels are elevated, and ECE activity is enhanced. Endothelin-1 is highly expressed in the lung and the over-activation of ET_A_ receptors and the reduced ET_B_ receptors activity result in intense vasoconstriction with matrix production and cell proliferation. These changes lead to fibrosis and inflammation of pulmonary artery and plasma values of ET-1 correlate with pulmonary vascular resistance levels [18].

Endotelin-1 secretion is inhibited by endothelium-derived relaxing factor, prostacyclin, atrial natriuretic peptide, and heparin [19, 20]. The plasma concentration of ET-1 in healthy subjects is very low, ranging between 1 and 2 pg/ml, levels that are below the threshold values ​for vasoconstriction effect. This excludes endocrine activity of ET-1 on target organs. On the contrary, the concentration of ET-1 due to autocrine/paracrine secretion at the level of the vascular endothelium appears to be higher, even if it has not been possible to prove this by direct measurements. It has been shown that the synthesis of ET-1 increases in some conditions of high cardiovascular risk such as hyperglycemia, hypercholesterolemia, arterial hypertension, estrogen deficiency, and aging [21]. Also, the plasma concentration of ET-1 tends to increase in the presence of cardiovascular diseases. Polymorphisms for the gene encoding ET-1 have been demonstrated; some of them appear to be associated with an increased risk of cardiovascular diseases such as arterial hypertension, angina pectoris, and acute coronary syndrome [22, 23].

## Endothelin ET-2

Endothelin-2 is mainly present in the ovary and intestine, but it has also been found in the vessels, heart, and kidney. Unlike ET-1, which is almost ubiquitous, ET-2 is not present in some organs and its concentration is approximately one-fifth of that of ET-1. Although the structures of ET-1 and ET-2 differ only in two amino acids, from a functional point of view, ET-2 appears to perform different actions than ET-1, and some of them are not yet fully explained. ET-2 may have an important activity in promoting ovulation and intestinal contraction, but it could also play a role in the process of formation of pulmonary alveoli and in thermoregulation. Animals that are experimentally deprived of ET-2 develop hypothermia, pulmonary emphysema, hypoglycemia, ketosis, and anemia [24].

## Endothelin ET-3

Endothelin-3 has a low affinity with ET_A_ receptors and therefore its activity is almost exclusively exerted through the ET_B_ receptors [25]. In mice, ET-3 was found in significant quantities in the brain, cerebellum, and medulla oblongata. This suggested that it was “the ET of the brain,” also because it was observed that in the human cerebral cortex about 90% of the endothelin receptors are ET_B_ receptors [5]. The ET-3/ET_B_ system is also relevant at the level of the autonomic nervous system, however, especially in the digestive system where it may have a role in controlling the motility, secretion, and perfusion of the intestine. ET-3 has also been detected in the placenta and kidney. While ET-3 can be found in human plasma, it is probably not produced by endothelial cells; the most likely source of plasma ET-3 is the adrenal gland [4]. Endothelin-3 may promote vasodilation through the release of NO and prostaglandins, even if at the vascular level, ET_B_ receptors are much less represented than ET_A_s. It has been shown that ET-3 may also have the ability to produce anti-inflammatory substances and promote the development of some cells such as melanocytes [11].

## Nitric oxide

Nitric oxide is a small molecule with important regulatory functions, including vasodilation, neurotransmission, and the regulation of genetic transcription [26]. Nitric oxide synthase (NOS) is the enzyme that catalyzes the formation of NO, starting from L-arginine and molecular oxygen, using reduced nicotinamide-adenine-dinucleotide phosphate, flavin adenine dinucleotide, flavin mononucleotide, and (6R-) 5,6,7,8-tetrahydro-L-biopterin. In mammals, NOS is produced in three different isoforms: neuronal NOS (nNOS), inducible NOS (iNOS), and endothelial NOS (eNOS) [27]. The nNOS is expressed in the brain where it has important functions in the mechanisms of learning, memory, and neurogenesis [28]. Neuronal NOS also plays a role in central blood pressure regulation. At the peripheral level, nNOS is present in the nitrergic nerves where it reduces the tone of smooth muscle cells. Inducible NOS is not produced in physiological conditions, but its production, mainly by macrophages, can be activated in inflammatory processes, in the presence of bacterial lipopolysaccharides, cytokines, and other agents [29]. Endothelial NOS is mainly produced in endothelial cells, where the highest concentration of this enzyme has been found, but eNOS has also been found in cardiac monocytes, platelets, placenta, and renal tubule cells. The production of NO at the endothelial level by eNOS, in addition to inducing vasodilation, may also have important anti-atherosclerotic actions. Endothelial NOS is activated by binding to calmodulin in the presence of an increased intracellular Ca^2+^ concentration. Through this physiological mechanism, the production of NO occurs in a pulsatile way. However, some stimuli, such as estrogen, vascular endothelial growth factor, insulin, bradykinin, or fluid shear stress, by phosphorylating some amino acids of the enzyme, activate eNOS in a more lasting way without the involvement of a significant increase in the entry of Ca^2+^ into the endothelial cells [30]. As previously mentioned, it has been found that the activation of ET_B_ receptors by ET is also a stimulus for the production of NO. The NOS system induces vasodilation of vessels of different sizes, through different mechanisms. In the larger arteries (conduit arteries), vasodilation is mediated by the action of NO on the soluble guanylate cyclase through activation of cGMP, while in the smaller arteries (resistance arteries), NO activates endothelium-derived hyperpolarizing factors through a series of reactions that result in the opening of Ca-dependent potassium channels, with consequent hyperpolarization of smooth muscle cells and vasodilation [31]. It should be remembered that, in addition to the NOS system, prostaglandins derived from arachidonic acid also contribute to vasodilation, both by promoting hyperpolarization processes and by activating cAMP.

**Fig. 2:**
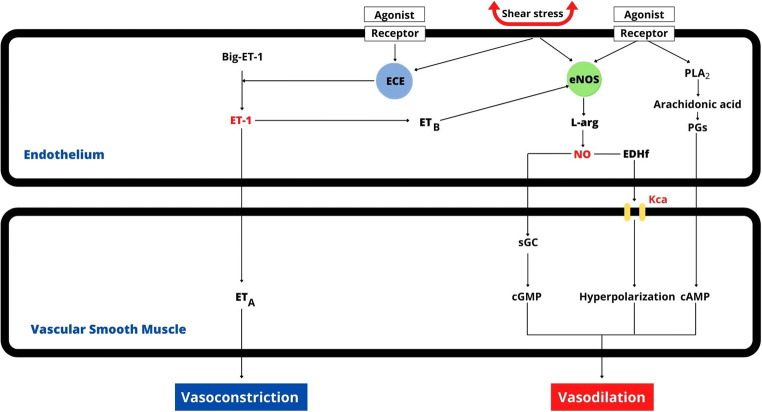
Balance between nitric oxide-mediated vasodilatory and ET-1-mediated vasoconstrictive activity. In larger arteries (conduit arteries), NO-dependent vasodilation is predominantly sGMP mediated, while EDH factors are more active in smaller arteries (resistance arteries). cAMP, cyclic adenosine monophosphate; cGMP, cyclic guanosine monophosphate; ECE, endothelin-converting enzyme; EDHf, endothelium-dependent hyperpolarization factors; eNOS, endothelial NO synthases; ET_A_/ET_B_, endothelin receptors; ET, endothelin; KCa, calcium-dependent potassium channels; L-arg, L-arginine; NO, nitric oxide; PGs, prostaglandins; PLA2, phospholipase A2; sGC, soluble guanylate cyclase

In conclusion, even if, given the extreme complexity of the system, the balance of vasoconstriction and vasodilation is not reducible to the mere relationship between ET-1 and NO, these two substances are the main players in this scenario (Fig. 2). The same ETs can have different effects depending on the receptor with which they interact, and both physical and chemical stimuli can lead to the activation of both vasoconstriction and vasodilation mechanisms through the ET/NO system [32]. Furthermore, in the presence of arterial hypertension, other mechanisms may interact with the endothelial system and the regulation of smooth muscle tone, including the autonomic nervous system and the renin-angiotensin-aldosterone system. A full discussion of this topic, however, is beyond the scope of this review. In children and adolescents, there is little evidence regarding the relationship between endothelial dysfunction and arterial hypertension. In any case, children could be ideal models to better understand the physiology of endothelial regulation of vascular tone and the pathogenesis of primary arterial hypertension, thanks to the lower presence of confounding factors due to aging, comorbidities, and other cardiovascular risk factors.

## Endothelin-1, nitric oxide, and hypertension in children and adolescents

Endothelin-1, given its proteic nature, can be measured directly in biological liquids. Whereas its plasma concentration is very low, below the threshold capable of exerting a vasoconstrictive activity, the autocrine/paracrine secretion at the level of the vascular endothelium is of greater physiological importance [7]. Substances that inhibit ET-1 activity, in particular receptor antagonists, have been widely used to study ET’s effects in vivo [33]. The damage of vascular endothelial function induced by increased plasma levels of ET-1 is one of the key factors in the pathogenesis of Idiopathic Pulmonary Arterial Hypertension and, although this is not part of the scope of our review, it is useful to remember that the use of ET receptor antagonists in this setting has greatly helped in understanding the interaction between ET and NO in humans [18]. While bosentan, a competitive antagonist of ET_A_ and ET_B_ receptors, has already been listed by the US Food and Drug Administration as an indication in children 3 years old and older with Idiopathic or Congenital Pulmonary Artery Hypertension [18], the use of this class of drugs for arterial hypertension should be limited to specific patients, such as adults with treatment-resistant hypertension or chronic kidney disease in whom the benefits outweigh the risks including the described hepatotoxicity, fluid retention, gonadal toxicity, and potential for teratogenicity [34]. Ongoing trials with ET receptor antagonists in focal glomerulosclerosis, IgA nephropathy, and resistant hypertension hold promise for an approved indication for ET receptor antagonists in treating kidney disease [35, 36]; nevertheless, their use in children is far from being considered for these indications.

The half-life of NO is extremely short, less than a second, as it is quickly converted to nitrite and/or nitrate by oxygen. Nitric oxide cannot be measured directly and its production can only be estimated from the concentrations of nitrite and nitrate end products [37]. In addition to this indirect method, the tool used most commonly in humans for the clinical assessment of the NO system function is the measurement of flow mediated dilation (FMD) at the level of the brachial artery [38]. The technique consists in measuring the increase in forearm blood flow following a 5-min occlusion of the brachial artery, through inflation of an arm cuff commonly used for blood pressure measurement, related to the baseline diameter of the artery. This vasodilation response is mainly mediated by the release of NO and is prevented by substances capable of selectively inhibiting eNOS [39].

Endothelial dysfunction is defined as “an imbalance between vasodilating and vasoconstricting substances produced by (or acting on) endothelial cells,” and is characterized by a reduction in vasodilation capacity and a pro-inflammatory and pro-thrombotic state [40]. There is evidence that the imbalance between NO and ET-1 plays an important role in the pathophysiology of essential hypertension in adults. The effects of various ET receptor antagonists on BP were evaluated in a meta-analysis including 4898 hypertensive patients, which showed a significant reduction of 24-h systolic and diastolic ambulatory BP associated with the administration of these drugs [41]. Also, attenuated NO bioavailability, the main characteristic of endothelial dysfunction, is present in adults with essential hypertension [42]. Moreover, clinical studies have shown that the arterial vasodilatory response to infusion of endothelium-dependent vasodilators in hypertensive patients is blunted, and that inhibition of NO leads to an increase in BP values [43].

To date, data on the interaction among ET-1, NO and hypertension in children and adolescents are limited. Some authors only evaluate the relationship between ET-1 and BP, while others only that between BP and NO values. There are very few studies that explore the ET-1/NO balance. A further problem in the interpretation of these studies is that often excess weight and high BP values coexist in children and it is known that, as early as childhood, the presence of obesity interferes with endothelial function.

It has been shown that plasma levels of ET-1 are significantly higher in children with excess weight [44] than in their normal weight peers. Furthermore, insulin resistance, often present in overweight and obese children and adolescents, may contribute to increased production of ET-1 [45]. Levels of ET-1 were found to be significantly higher in a group of Polish adolescents with several cardiovascular risk factors (obesity and/or hypertension and/or diabetes) than in a control group [46]. In a population of adolescents (mean age 16.5 years), Katona et al. found significantly higher plasma ET-1 and significantly lower NO values in hypertensive individuals as compared to normotensive controls. Interestingly, there was a significant difference in body weight between the two groups. The study also showed a positive correlation between the plasma concentrations of ET-1 and systolic BP values, while an inverse correlation was observed for NO [47]. Similar results are described by Aflyatumova et al. in adolescents (average age 16 years) in which ET-1 values progressively and significantly increase together with worsening of the BP category (going from normotension to pre-hypertension and then to hypertension). Plasma NO values were significantly lower in the hypertensive than in the normotensive individuals, while body mass index (BMI) values were not different in the compared groups [48]. Banaszak et al. found that, compared to controls, ET-1 levels were significantly higher in hypertensive adolescents (13.6 years old) with both primary and kidney hypertension. Body mass index was higher in subjects with primary hypertension than in both control and kidney hypertension groups [49]. However, the association between hypertension and high ET-1 was not confirmed by a recent Italian study, which, while demonstrating a close relationship between elevated ET-1 values and excess weight in 11-year old children, did not find any differences in ET-1 concentration between normotensive and hypertensive individuals of the same weight class. In this study, unlike what was described by Katona et al., NO levels were directly and independently correlated to diastolic BP, even after adjustment for BMI and insulin resistance [50].

A series of studies investigated only the NO system, without evaluating ET-1. In a large population of pre-pubertal children, obesity was associated with higher FMD, and lower arterial stiffness, despite the presence of higher BP values [51]. In agreement, a Portuguese study performed in children aged 8–9 years found that urinary nitrites and nitrates were increased in both overweight and obese children independently of ABPM results [52]. Similar findings have been described in a population of Chinese children, in which those who were overweight or obese displayed significantly higher levels of NO end-products than those with normal weight. However, blood pressure values were not reported in this study [53]. On the contrary, in older obese individuals (mean age 14 years), Gruber et al. showed a decreased bioavailability of NO compared with normal weight controls. Moreover, systolic BP values were significantly higher in the excess weight group [54]. These findings suggest an adaptive response in early stages of obesity which may lead to endothelial dysfunction later in life.

Contradictory results emerging from the studies described above may at least partly depend on the age of the study subjects. In fact, it seems that, in the presence of obesity and hypertension, the ET-1/NO imbalance in younger children is less pronounced than in adolescents. In particular, there seems to be an overstimulation, rather than a reduction, of NO levels and of its effects associated with increased BP levels in the lower age groups, whereas in hypertensive adolescents, ET-1/NO imbalance would be similar to what is described in adults. Overall, an important role of body weight in determining the plasma concentration of ET-1 is evident.

From the available studies, it is not easy to understand whether it is the presence of arterial hypertension that determines an alteration in the ET-1/NO balance or whether the imbalance between ET and NO precedes the development of hypertension.

**Fig. 3:**
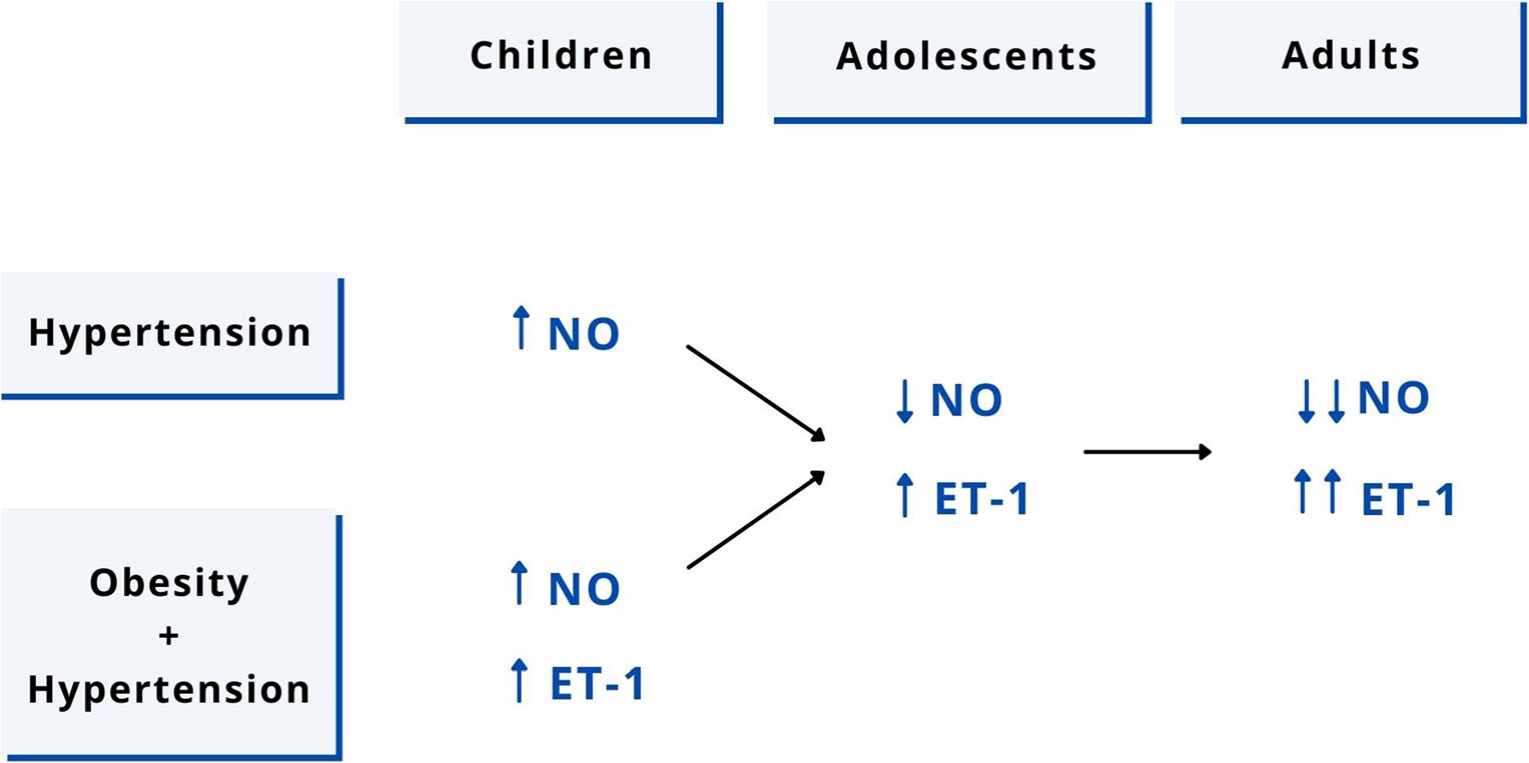
Hypotheses about NO and ET-1 changes in the onset and maintenance of hypertension from childhood to adulthood. NO, nitric oxide; ET-1, endothelin-1

As previously described, it would seem that, at least in obese children with increased BP values, the response induced by ischemic stress is greater than in normotensive individuals, suggesting that endothelial production of NO is overstimulated in the early phases of hypertension, when there is a significant excess of weight. A similar phenomenon has also been described in adolescents with elevated BP values who had a low birth weight [55]. This phenomenon could represent a tentative protective mechanism that is active before the development of a stable hypertension condition. The Cardiovascular Risk in Young Finns Study demonstrated that the presence of elevated systolic BP in adolescence predicts impaired brachial endothelial function in adulthood [56]. These data support the hypothesis that in hypertensive children, endothelial dysfunction is the consequence, rather than the cause, of the increase in BP. However, it is also possible to hypothesize that a certain degree of alteration of endothelial function (increased FMD due to NO hypersecretion) emerges in parallel with hypertension development. In the initial stages of hypertension, when organ damage has not yet had time to develop, endothelial production of NO is chronically overstimulated. This pathophysiological condition may initially protect young vessels from the development of stable hypertension. In a second step, a decrease in the synthesis and release of NO causes endothelial dysfunction and, associated with an increase in ET-1, favors early damage to the vascular wall in obese and/or hypertensive young children (Fig. 3). Further longitudinal studies in large populations allowing stratification of children and adolescents by age, weight status, and presence/absence of hypertension are needed to clarify the complex relationship between NO and ET-1 and to confirm this pathophysiological model for arterial hypertension and subclinical organ damage.

## Key summary points

- A nitric oxide/endothelin-1 imbalance plays a role in the pathophysiology of essential hypertension in adults, in which there is a reduction in nitric oxide activity and an increase in that of endothelin-1.
- Regarding hypertensive children, it is not possible to reach definitive conclusions due to the limited number of studies available and the presence of confounding factors, such as the difference in age, pubertal development, and weight class in the cohorts included in available studies.
- Several studies show that in children and adolescents, excess weight and/or arterial hypertension are associated with an increase in endothelin-1 values.
- pt?>In younger subjects, in the presence of obesity and/or arterial hypertension, the nitric oxide system seems to be over-expressed, but over time, this condition would be followed by a reduction in the bioavailability of nitric oxide.
- Further studies are needed to understand the complex relationship between nitric oxide, endothelin-1, and arterial hypertension in children and adolescents.

## Multiple-choice questions (answers are provided following the reference list)

1. Which endothelin activates endothelin_A_ receptors?

- Only endothelin-1
- Only endothelin-2
- Only endothelin-3
- Both endothelin-1 and endothelin-2
- Endothelin-1, endothelin-2 and endothelin-3 all activate endothelin_A_ receptors

2. Which of these substances does not stimulate the synthesis of endothelin-1?

- Thrombin
- TNF-α
- Heparin
- Insulin
- Vasopressin

3. Which of these statements is incorrect?
  - To be active, the nitric oxide synthase enzyme requires all these cofactors: NADH, FAD, FMN, BH4.
  - The nitric oxide synthase enzyme is stimulated by endothelin-3.
  - Endothelin-3 stimulates vasodilation through cyclic GMP and endothelium-derived hyperpolarizing factors.
  - The nitric oxide synthase enzyme is always active in the central nervous system.
  - Estrogen stimulates the production of nitric oxide synthase enzyme.
4. How is endothelial dysfunction defined?

- An imbalance between vasodilating and vasoconstricting substances produced by muscle cells
- An imbalance between vasodilating and vasoconstricting substances produced by endothelial cells
- An increase in vasodilation capacity
- A decrease of pro-inflammatory and pro-thrombotic state
- An increase of nitric oxide bioavailability and a decrease of endothelin-1 plasma values

5. The balance of nitric oxide and endothelin-1 in hypertensive children:

- is similar to that described in hypertensive adults
- is similar to that described in hypertensive adults only regarding the bioavailability of nitric oxide
- is influenced by the presence of excess weight
- is always characterized by an increase in plasma endothelin-1 values
- has been studied predominantly in prepubescent children and there is a lack of evidence in adolescents
